# Water and Fertilizer Management Is an Important Way to Synergistically Enhance the Yield, Rice Quality and Lodging Resistance of Hybrid Rice

**DOI:** 10.3390/plants13172518

**Published:** 2024-09-07

**Authors:** Haijun Zhu, Lingli Nie, Xiaoe He, Xuehua Wang, Pan Long, Hongyi Chen

**Affiliations:** 1 Key Laboratory of Ministry of Education for Crop Physiology and Molecular Biology, Hunan Agricultural University, Changsha 410128, China; 15211104718@163.com (H.Z.); linglinie@163.com (L.N.); hexiaoe2022@163.com (X.H.); 2 State Key Laboratory of Hybrid Rice, Hunan Hybrid Rice Research Center, Changsha 410125, China

**Keywords:** yield, quality, lodging resistance, NSC, fertilizer management, irrigation methods

## Abstract

This study comprehensively investigated the synergistic effects and underlying mechanisms of optimized water and fertilizer management on the yield, quality, and lodging resistance of hybrid rice (*Oryza sativa*), through a two-year field experiment. Two hybrid rice varieties, Xinxiangliangyou 1751 (XXLY1751) and Yueliangyou Meixiang Xinzhan (YLYMXXZ), were subjected to three irrigation methods (W1: wet irrigation, W2: flooding irrigation, W3: shallow-wet-dry irrigation) and four nitrogen fertilizer treatments (F1 to F4 with application rates of 0, 180, 225, and 270 kg ha^−1^, respectively). Our results revealed that the W1F3 treatment significantly enhanced photosynthetic efficiency and non-structural carbohydrate (NSC) accumulation, laying a robust foundation for high yield and quality. NSC accumulation not only supported rice growth but also directly influenced starch and protein synthesis, ensuring smooth grain filling and significantly improving yield and quality. Moreover, NSC strengthened stem fullness and thickness, converting them into structural carbohydrates such as cellulose and lignin, which substantially increased stem mechanical strength and lodging resistance. Statistical analysis demonstrated that water and fertilizer treatments had significant main and interactive effects on photosynthetic rate, dry matter accumulation, yield, quality parameters, NSC, cellulose, lignin, and stem bending resistance. This study reveals the intricate relationship between water and fertilizer management and NSC dynamics, providing valuable theoretical and practical insights for high-yield and high-quality cultivation of hybrid rice, significantly contributing to the sustainable development of modern agriculture.

## 1. Introduction

Water and fertilizer management has always occupied a crucial position in the agricultural field, especially for hybrid rice (*Oryza sativa*), and its significance is even more far-reaching and significant [1]. In the current context where the global food security situation is becoming increasingly severe, continuously increasing the yield of rice to meet the huge demand for food from the continuously growing population has become an urgent and crucial task [2]. For hybrid rice, as a crop type with significant yield-increasing potential, the significance of effectively enhancing its yield to ensure an adequate supply of food goes without saying [3,4]. Numerous past research results clearly indicate that a reasonable and precise water and fertilizer management model can have a significant positive impact on the yield of hybrid rice [5,6,7]. Through in-depth research, Zhu [1] found that precisely regulating the irrigation water volume and the time, type, and dosage of fertilizer application can create the most suitable growth environment for hybrid rice, thereby greatly promoting the efficiency of photosynthesis and the effect of nutrient absorption, laying a solid foundation for a substantial increase in yield.

However, the goal we pursue is by no means limited solely to the simple increase in yield. With the development of modern society and the significant improvement of people’s living standards, strict requirements for the quality of rice have also gradually emerged [8]. High-quality rice is not only closely related to the enjoyment of taste and the level of nutritional value, but also directly related to people’s health status [9]. Water and fertilizer management play a vital role in influencing the quality of rice [10]. The just-right water and fertilizer supply model can finely regulate many aspects of rice, including appearance quality, processing quality, and cooking and taste quality [11,12]. The research of Sun [13] clearly pointed out that a specific water management method can effectively reduce the chalkiness degree and thereby significantly improve the rate of whole milled rice, while Ning [14], through a large number of experiments and data analysis found that a reasonable fertilizer operation plan can effectively improve the protein content and starch characteristics of rice, thereby optimizing its eating quality. At the same time, the lodging of hybrid rice has long been one of the troublesome problems that have plagued the majority of growers [15]. Lodging will not only directly lead to a serious loss of yield, but also bring great difficulties and cost increases to the harvesting work, and have a serious impact on the economic benefits of rice production [16]. Furthermore, the scientific and reasonable water and fertilizer management strategy can effectively enhance the stem strength and lodging resistance ability of rice plants. By rationally regulating moisture and avoiding long-term excessive water logging or severe drought conditions, it can strongly promote the good development of the root system and the robust growth of the stem [17]. In terms of fertilizer management, achieving a balanced supply of various nutritional elements such as nitrogen, phosphorus, and potassium, especially strictly controlling the excessive application of nitrogen fertilizer, plays a crucial role in enhancing the mechanical strength and toughness of the stem [18]. Many related research works such as Zhang’s [19] have deeply revealed the close internal connection existing between water and fertilizer management and lodging resistance.

In the past abundant research process, many scholars have carried out extensive and in-depth explorations on the relationship between water and fertilizer management and the yield, quality, and lodging resistance of hybrid rice [20,21,22]. However, despite the fruitful research results that have been achieved, there is still a series of key problems that urgently need to be further deeply studied and properly solved. How to accurately formulate personalized water and fertilizer management plans for different ecological environments and planting conditions to achieve the best synergistic effect of yield, quality, and lodging resistance is still a highly challenging topic. In addition, regarding the internal mechanism of how water and fertilizer management affects the physiological and biochemical processes of hybrid rice, our current understanding still has certain limitations and requires more in-depth and comprehensive research to fully reveal its internal laws.

This study aims to deeply and systematically explore the relationship between water and fertilizer management and the yield, quality, and lodging resistance of hybrid rice. By comprehensively using diversified research methods such as field experiments and physiological and biochemical analyses, it strives to fully reveal the internal mechanism and synergistic effect. We earnestly aim that through this study, we can provide a solid scientific basis and practical operational guidance for optimizing the water and fertilizer management of hybrid rice, strongly promoting the sustainable development of the hybrid rice industry, and making due contributions to effectively ensuring global food security and significantly improving people’s quality of life. Meanwhile, our goal is also that this study can attract more scholars’ high attention and in-depth discussion in this field, thereby promoting the continuous progress and development of relevant research.

## 2. Results

### 2.1. Photosynthetic Rate

**Figure 1:**
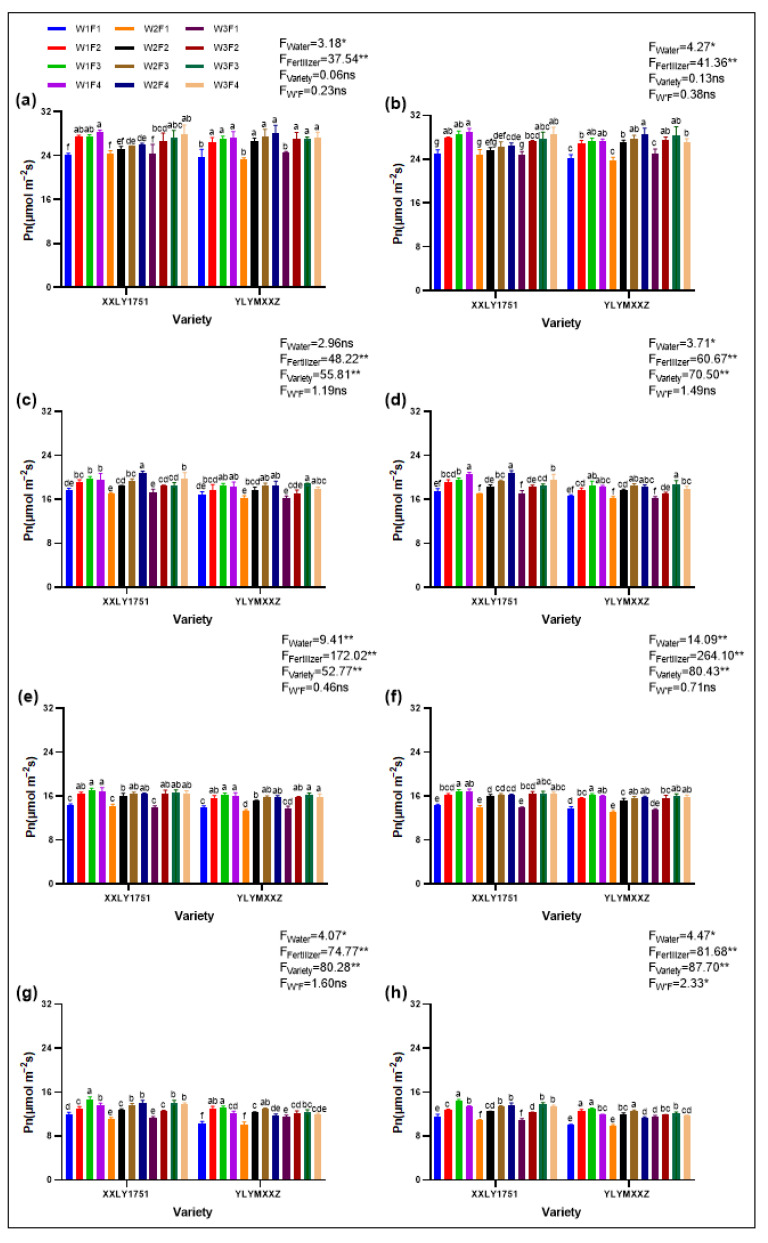
(**a**,**b**) represent the tillering stages in 2022 and 2023, respectively; (**c**,**d**) represent the booting stages in 2022 and 2023, respectively; (**e**,**f**) represent the full heading stages in 2022 and 2023, respectively; (**g**,**h**) represent the grain filling stages in 2022 and 2023, respectively. Different lowercase letters on the error bars denote statistical differences (at the 0.05 level) among treatments of various varieties based on the LSD test. Significant differences within the same treatment are denoted by ns (*p* > 0.05), * (0.01 < *p* ≤ 0.05), and ** (*p* ≤ 0.01).

Water and fertilizer management has a significant impact on the net photosynthetic rate of leaves during different growth stages of hybrid rice (Figure 1)**.** Among them, different irrigation treatments had a significant effect (*p* < 0.05) on the transpiration rate of rice leaves during the tillering and grain filling stages in 2022, as well as the tillering, booting, and grain filling stages in 2023. There was no significant effect during the booting stage in 2022, but there was a highly significant effect (*p* < 0.01) during other growth stages. Fertilizer had a highly significant effect (*p* < 0.01) on the photosynthetic rate during all examined periods. Varietal differences had no significant effect on the net photosynthetic rate during the tillering stage in both 2022 and 2023, but had a highly significant effect (*p* < 0.01) during other growth stages. The interaction between water and fertilizer only had a significant effect (*p* < 0.05) during the grain filling stage in 2023.

As rice grows, the photosynthetic rate shows a downward trend, with the highest rate during the tillering stage and the lowest during the grain filling stage. The water treatment strategy varies at different growth stages: from tillering to booting, W3 is the most optimal; from full heading to grain filling, W1 is better. The effect of fertilizer application also varies by stage: from tillering to booting, the photosynthetic rate increases with increasing fertilizer application, i.e., F4 > F3 > F2 > F1; from full heading to grain filling, it first increases and then decreases, i.e., F3 > F4 > F2 > F1.

There are significant differences in the net photosynthetic rate of rice leaves under different water, fertilizer, and cultivar treatments. Taking the full heading stage as an example, Xinxiangliangyou 1751 had the highest net photosynthetic rate under W1F4V1 treatment in both 2022 and 2023, with rates of 17.01 µmol m^−2^ s^−1^ and 16.89 µmol m^−2^ s^−1^, respectively. For Yueliangyou Meixiangxinzhan, the highest net photosynthetic rate during the full heading stage in both 2022 and 2023 was achieved under W1F3V1 treatment, with rates of 16.29 µmol m^−2^ s^−1^ and 16.18 µmol m^−2^ s^−1^, respectively.

### 2.2. Total Dry Weight (TDW)

**Figure 2:**
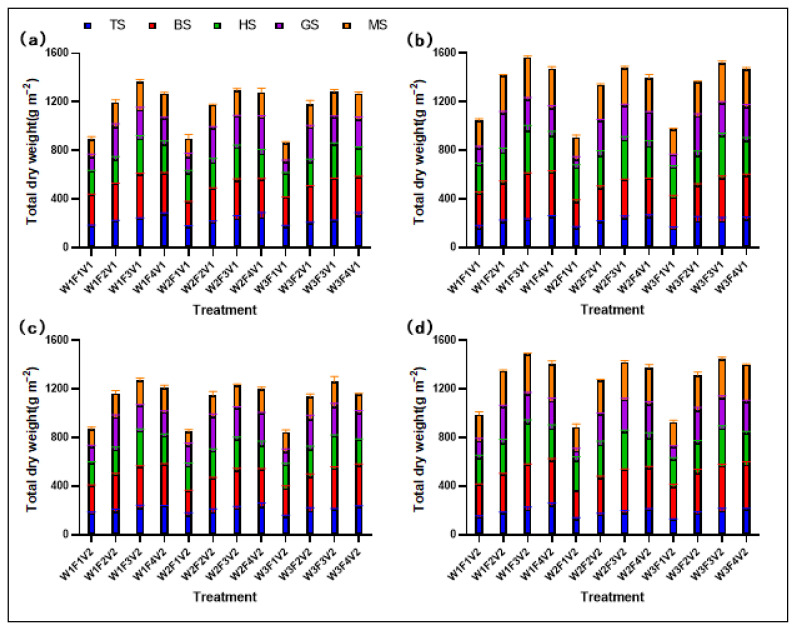
TDW of XXLY1751 (**a**) and YLYMXXZ (**b**) in 2022, and XXLY1751 (**c**) and YLYMXXZ (**d**) in 2023.

Distinct differences in TDW accumulation were observed across rice growth stages under diverse water and fertilizer treatments (Figure 2). Treatments W1F3V1 and W1F3V2 were identified as the most effective in promoting TDW accumulation, achieving above-ground dry matter levels of 1465.19 g m^−2^ and 1377.51 g m^−2^ at maturity, respectively, significantly surpassing other treatment groups. These findings unequivocally demonstrate that appropriate water and fertilizer management strategies can significantly enhance the dry matter accumulation of rice, potentially leading to positive impacts on its yield and quality.

### 2.3. Yield

**Figure 3:**
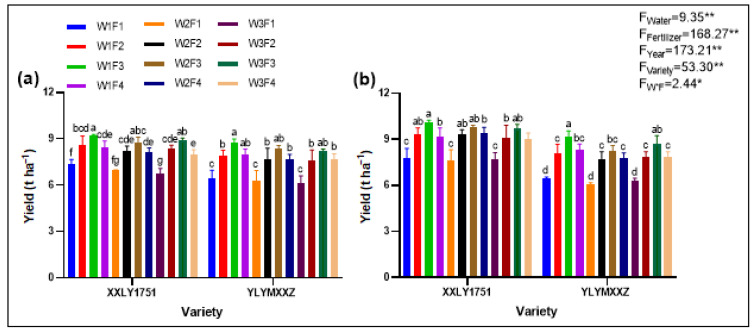
Figures (**a**,**b**) represent the yields of Xinxiangliangyou 1751 and Yueliangyou Meixiangxinzhan in 2022 and 2023, respectively. Different lowercase letters on the error bars indicate statistical differences (at a significance level of 0.05) between treatments of various cultivars in the LSD test. Significant differences within the same treatment are denoted by * (0.01 < *p* ≤ 0.05), and ** (*p* ≤ 0.01).

The yield of rice was significantly influenced by water and fertilizer management strategies (Figure 3). Treatments W1F3V1 and W1F3V2 demonstrated the highest yields, averaging 9.64 t ha^−1^ and 8.99 t ha^−1^, respectively, while W2F1V1 and W2F1V2 resulted in the lowest yields. The significant differences in yields among treatment groups highlight the effectiveness of optimized water and fertilizer management in enhancing rice productivity.

### 2.4. Appearance Quality

**Figure 4:**
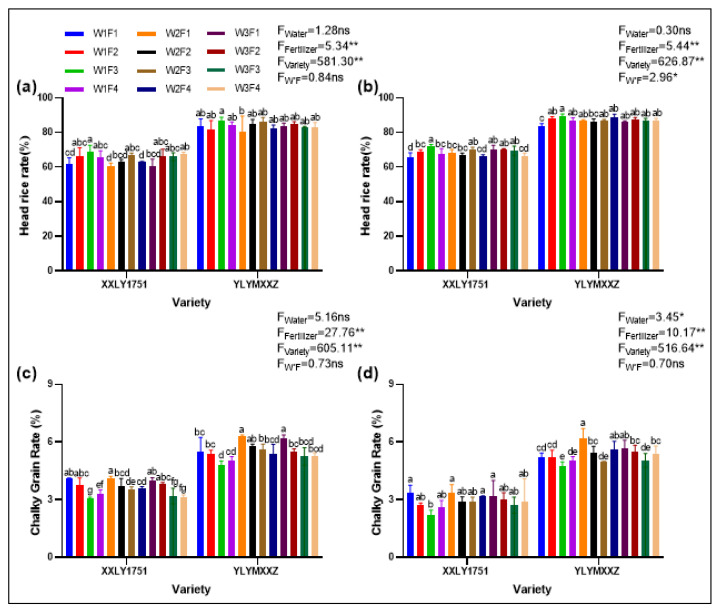
Head rice rate of XXLY1751 and YLYMXXZ in 2022 (**a**) and 2023 (**b**), and chalky grain rate of XXLY1751and YLYMXXZ in 2022 (**c**) and in 2023 (**d**). Different lowercase letters denote statistical differences between treatments of each season according to the LSD test (0.05). Significant differences within the same treatment are denoted by ns (*p* > 0.05), * (0.01 < *p* ≤ 0.05), and ** (*p* ≤ 0.01).

Water and fertilizer management strategies had significant effects on the appearance quality of rice, as evidenced by variations in the head rice rate and chalky grain rate (Figure 4). Treatments W1F3V1 and W1F3V2 resulted in higher head rice rates and lower chalky grain rates compared to other treatments, indicating improvements in both processing and appearance qualities. Specifically, the head rice rate reached 70.4% under treatment W1F3V1, while treatment W1F3V2 achieved an impressive 88.3%, both of which were significantly higher than other treatment groups. However, in terms of chalky grain rate, the W1F3V1 and W1F3V2 treatments showed relatively lower performance. The data reveal that the chalky grain rate was 3.2% for W1F3V1 and 4.8% for W1F3V2, both significantly lower than other treatments. The finding indicates that through careful water and fertilizer management, the head rice rate can be effectively increased while reducing the chalky grain rate, thereby enhancing the overall quality of rice.

### 2.5. Nutritional Quality

**Figure 5:**
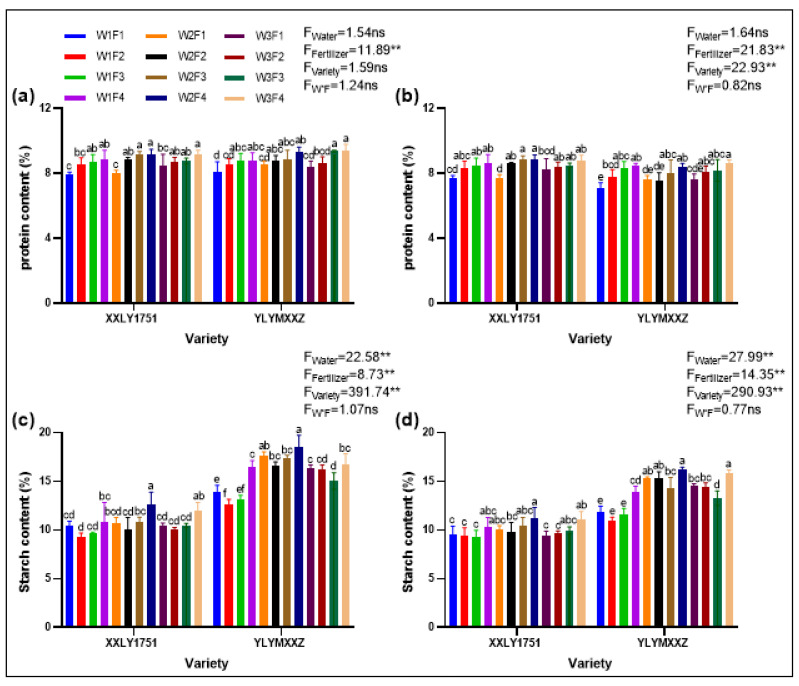
Protein content of XXLY1751 and YLYMXXZ in 2022 (**a**) and 2023 (**b**), and amylose content of XXLY1751and YLYMXXZ in 2022 (**c**) and 2023 (**d**). Different lowercase letters denote statistical differences between treatments of each season according to the LSD test (0.05). Significant differences within the same treatment are denoted by ns (*p* > 0.05), and ** (*p* ≤ 0.01).

The effects of water and fertilizer management on protein and amylose content in different high-quality hybrid rice varieties are shown in the accompanying figures (Figure 5). After thorough analysis, we have drawn the following conclusions: Regarding protein content, irrigation treatments did not significantly impact it in 2022 and 2023. However, fertilizer treatments had a highly significant effect (*p* < 0.01), showing an increasing trend with higher fertilizer application, where the F4 treatment resulted in the highest protein content. The cultivar treatment had a significant influence on protein in 2023 (*p* < 0.05), but no notable difference was observed in 2022. It is worth mentioning that the interaction between water and fertilizer did not significantly affect protein content. In terms of specific cultivar responses, Xinxiangliangyou 1751 had the highest protein content under the W2F4V1 treatment, while Yueliangyou Meixiangxinzhan led under the W3F4V2 treatment.

As for amylose content, irrigation, fertilizer, and cultivar treatments all had a highly significant impact (*p* < 0.01) in both years. Wet irrigation (W1 treatment) generally effectively reduced amylose content. The effect of fertilizer treatments showed varying patterns, particularly in the case of Xinxiangliangyou 1751 in 2022, where the amylose content decreased initially with increasing fertilizer application and then rose, with the F3 treatment resulting in the lowest content. Similarly, the interaction between water and fertilizer did not significantly affect amylose content. Regarding cultivar responses, Xinxiangliangyou 1751 had the highest amylose content under the W1F2V1 treatment, while Yueliangyou Meixiangxinzhan led under the W1F2V2 treatment.

### 2.6. Plant Height at Center of Gravity and Panicle Length

**Figure 6:**
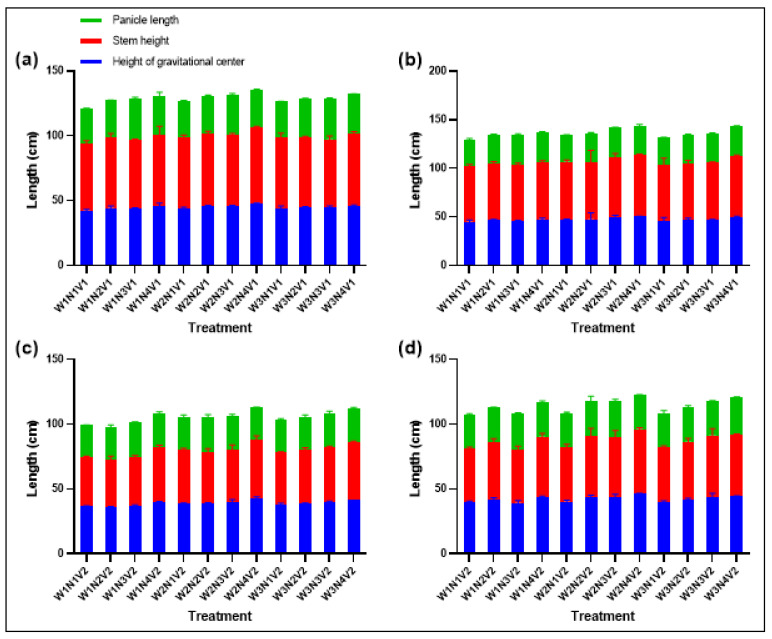
Plant height at center of gravity and panicle length of XXLY1751 (**a**) and YLYMXXZ (**b**) in 2022, and XXLY1751 (**c**) and YLYMXXZ (**d**) in 2023.

Irrigation and fertilization strategies significantly affected plant height at the center of gravity and panicle length. W1F3V1 and W1F3V2 treatments resulted in longer panicles, indicating the potential of optimized water and fertilizer management in promoting rice panicle growth (Figure 6). Regarding plant height at the center of gravity, distinct differences were observed among different irrigation treatments, with an overall trend of W2 > W3 > W1. Meanwhile, various fertilization treatments also exhibited a certain pattern of influence on plant height at the center of gravity, specifically F4 > F2 > F3 > N1. These results indicate that irrigation and fertilization strategies have a significant regulatory effect on the plant height at the center of gravity of rice plants. In terms of panicle length, the W1F3V1 and W1F3V2 treatments demonstrated notable advantages. Specifically, the panicle length of the W1F3V1 treatment reached 31.46 cm, while that of the W1F3V2 treatment was 27.70 cm.

### 2.7. Lodging Index

Irrigation and fertilization treatments demonstrated significant effects on rice lodging resistance (Table 1). The F3 treatment was identified as the most effective in enhancing stem bending resistance and lodging index, while the W1 treatment also showed a positive impact on lodging resistance. Regarding the influence of irrigation treatments on bending resistance, we observed a general trend of W1 > W3 > W2. However, this trend is reversed when considering the lodging index. This finding indicates that wet irrigation treatment (i.e., W1 treatment) has a significant effect on enhancing rice lodging resistance. Therefore, we can conclude that both W1F3 and W1F2 treatments are effective regulatory measures to improve rice lodging resistance.

### 2.8. Non-Structural Carbohydrate (NSC)

**Figure 7:**
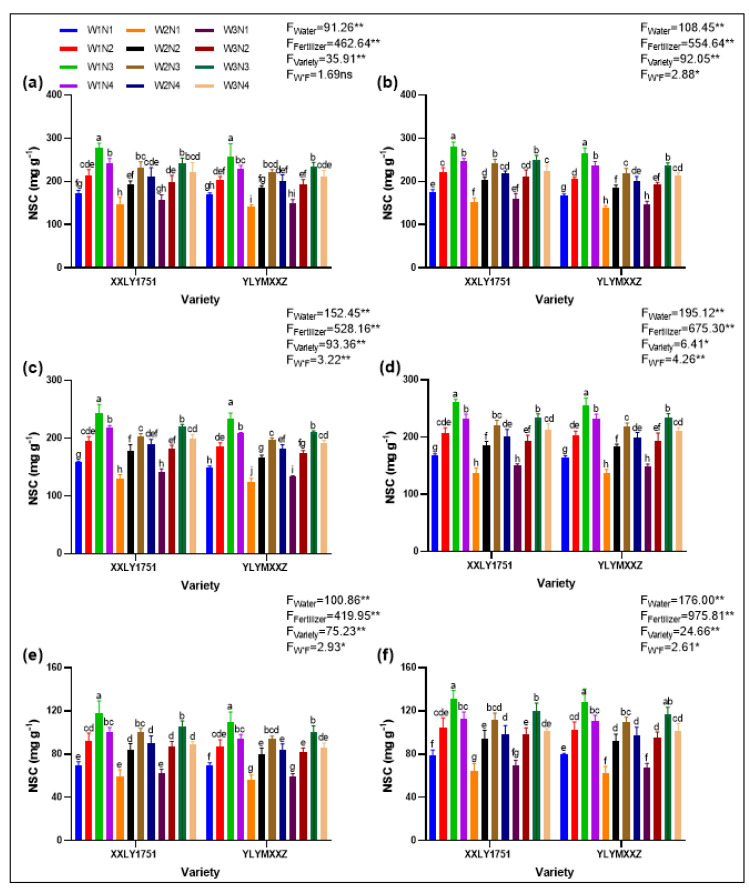
(**a**,**b**) represent the full heading stages in 2022 and 2023, respectively; (**c**,**d**) represent the grain filling stages in 2022 and 2023, respectively; (**e**,**f**) represent the mature stage in 2022 and 2023, respectively. Different lowercase letters on the error bars denote statistical differences (at the 0.05 level) among treatments of various varieties based on the LSD test. Significant differences within the same treatment are denoted by ns (*p* > 0.05), * (0.01 < *p* ≤ 0.05), and ** (*p* ≤ 0.01).

NSC content in rice stems was influenced by water and fertilizer management (Figure 7). The W1F3V1 and W1F3V2 treatments significantly increased NSC content during the grain-filling period, suggesting a positive effect of optimized water and fertilizer management on NSC accumulation. From the heading stage to maturity, we observed a continuous decline in stem NSC content across all treatment groups. This indicates that NSC in the stems is gradually transported to the panicle to support its growth and grain filling. Taking the grain-filling period as an example, we further analyzed the impact of different treatments on stem NSC content. The results showed that the stem NSC content of the W1F3V1 and W1F3V2 treatments was significantly higher than that of other treatment groups, reaching 230.93 mg g^−1^ and 222.20 mg g^−1^, respectively. This suggests that water and fertilizer management can effectively increase the stem NSC content during the grain-filling period of rice, which may positively impact rice yield and quality.

### 2.9. Lignin and Cellulose

**Figure 8:**
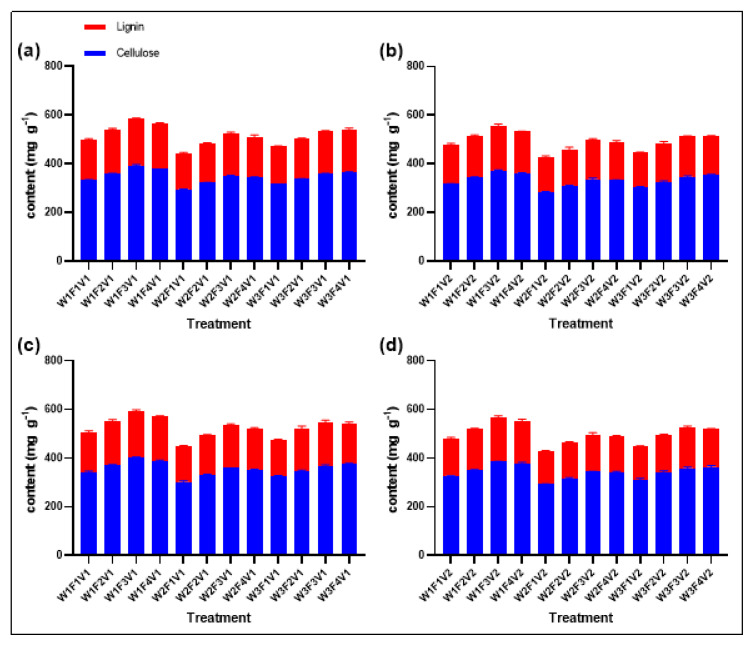
Lignin and cellulose of XXLY1751 (**a**) and YLYMXXZ (**b**) in 2022, and XXLY1751 (**c**) and YLYMXXZ (**d**) in 2023. Different lowercase letters denote statistical differences between treatments of each season according to an LSD test (0.05).

Water and fertilizer management significantly affected the structural carbohydrate content of rice stems (Figure 8). According to comprehensive data from both years, the W1F3V1 and W1F3V2 treatments exhibited clear advantages in stem cellulose content. Specifically, the stem cellulose content in the W1F3V1 treatment was 398.11 mg g^−1^, which was 15.01 to 99.4 mg g^−1^ higher than other treatment groups. Similarly, the content in the W1F3V2 treatment was 378.74 mg g^−1^, exceeding other treatments by 19.28 to 89.68 mg g^−1^. Likewise, in terms of stem lignin content, the W1F3V1 and W1F3V2 treatments also led significantly. The lignin content in W1F3V1 was 191.58 mg g^−1^, which was 5.42 to 44.96 mg g^−1^ higher than other treatments. For W1F3V2, the content was 181.85 mg g^−1^, surpassing other treatment groups by 8.23 to 44.09 mg g^−1^. These findings suggest that specific water and fertilizer management strategies, such as the W1F3V1 and W1F3V2 treatments, can significantly increase the structural carbohydrate content in rice stems. This may positively impact the mechanical strength and lodging resistance of the stems.

### 2.10. Amylase Activity

**Figure 9:**
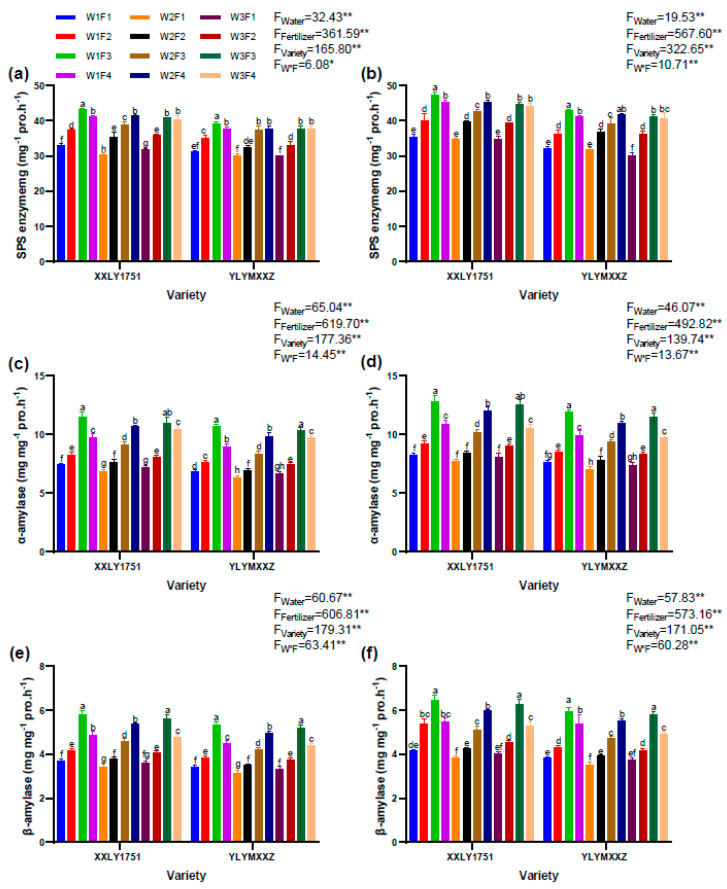
SPS enzyme activity of XXLY1751 and YLYMXXZ in 2022 (**a**) and in 2023 (**b**), α-amylase activity of XXLY1751 and YLYMXXZ in 2022 (**c**) and in 2023 (**d**),and β-amylase activity of XXLY1751 and YLYMXXZ in 2022 (**e**) and in 2023 (**f**). Different lowercase letters denote statistical differences between treatments of each season according to an LSD test (0.05). Significant differences within the same treatment are denoted by * (0.01 < *p* ≤ 0.05), and ** (*p* ≤ 0.01).

The activities of SPS enzyme, α-amylase, and β-amylase in rice stems were influenced by water and fertilizer management (Figure 9). Upon comprehensive comparison of the experimental outcomes from both years, it was observed that during the grain filling period, the contents of SPS enzyme, α-amylase, and β-amylase in the stems under the W1F3V1 and W1F3V2 treatments were notably higher than those in other treatment groups. Specifically, the contents of SPS enzyme, α-amylase, and β-amylase in the W1F3V1 treatment were 45.12 mg g^−1^, 12.16 mg g^−1^, and 6.11 mg g^−1^, respectively, while those in the W1F3V2 treatment were 41.03 mg g^−1^, 11.20 mg g^−1^, and 5.63 mg g^−1^, respectively. The W1F3V1 and W1F3V2 treatments were associated with higher enzyme activities, suggesting a positive impact on carbohydrate metabolism.

### 2.11. Variance Analysis

We have discovered that both water and fertilizer treatments exert significant influences on the physiological indicators, yield, and quality of rice (Table 2). More specifically, these two treatments serve as primary effects, effectively enhancing the photosynthetic rate, dry matter accumulation during maturity, yield, head rice rate, as well as the protein and amylose content in rice grains. Concurrently, there is a notable increase in the content of NSC, cellulose, lignin, and the bending resistance of the stems.

Further data analysis has unveiled significant interactive effects between water and fertilizer on various aspects of rice, including the photosynthetic rate, dry matter accumulation during maturity, yield, head rice rate, amylose content in rice grains, cellulose content in stems, and stem bending resistance. It is worth noting that this interaction demonstrates consistency in its impact on two rice varieties across two years of experimentation, indicating that there is no significant interaction between water, fertilizer, and rice varieties. Similarly, no apparent interaction was observed between water, fertilizer, and the experimental year. These findings strongly suggest that when water management and fertilizer application strategies are appropriately combined, they can lead to a more substantial improvement in the aforementioned physiological, yield, and quality indicators.

## 3. Discussion

### 3.1. Effect of Water and Fertilizer on the Yield

The application of optimized water and fertilizer management strategies in this study, specifically the W1F3 treatment, has demonstrated significant effectiveness in boosting rice yield. This approach notably improved photosynthetic efficiency, which is critical for biomass accumulation and grain production in rice [1]. By enhancing photosynthesis through precise regulation of water and nitrogen, the vegetative organs of the rice were strengthened, the leaf area was increased, and leaf senescence was delayed. These physiological changes led to improved carboxylation efficiency of CO₂ in mesophyll cells, thereby increasing the rate of CO₂ assimilation [4]. This series of physiological responses not only established a solid material foundation for high rice yields but also reflected the excellent efficacy of the W1F3 treatment in optimizing rice’s physiological functions.

The elevated photosynthetic rates resulting from the W1F3 treatment supported healthy growth in rice plants, leading to improved dry matter production efficiency. This increase in dry matter accumulation directly contributed to building a productive rice population [23]. Additionally, efficient transfer of NSC stored in rice stems and sheaths during the grain-filling phase is essential for determining the final yield. By promoting NSC accumulation and refining their transfer mechanism, the W1F3 treatment further optimized the yield formation process [24,25].

In conclusion, the W1F3 treatment enhanced yield through various mechanisms: improving photosynthetic efficiency, facilitating dry matter accumulation, and optimizing NSC dynamics. These results offer valuable insights into the potential for increasing rice yield and provide new perspectives for achieving high-yield rice cultivation in the future.

### 3.2. Effect of Water and Fertilizer on Quality

The application of refined water and fertilizer management tactics, especially the W1F3 treatment, has exhibited notable impacts on elevating the quality of hybrid rice. Through meticulous regulation of the growth conditions, this method not only augments photosynthetic efficiency but also substantially affects carbohydrate metabolism and nutritional makeup.

The boosted photosynthetic efficiency resulting from the W1F3 treatment leads to an amplified accumulation of NSC, encompassing soluble sugars and starches. These NSC constitute a vital material foundation for starch synthesis and protein formation in rice grains, thereby fostering enhanced rice quality [26]. Specifically, the W1F3 treatment guarantees seamless grain filling, giving rise to fuller grains and an overall elevation in the aesthetic quality of rice. This improvement is manifested in the considerable augmentation of the head rice rate and grain length–width ratio, signifying advancements in both processing and visual attributes [24,27].

Furthermore, the W1F3 treatment brings about a diminished chalkiness degree and chalky grain rate, further underscoring its proficiency in upgrading rice quality. Chalkiness, a pivotal aspect influencing rice quality, is intimately linked to carbohydrate metabolism and nutrient absorption [28]. The optimized water and fertilizer management inherent in the W1F3 treatment adeptly regulates these procedures, consequently minimizing chalkiness and elevating rice quality.

In essence, the W1F3 treatment, via its thorough optimization of the rice growth milieu, considerably boosts photosynthetic efficiency and carbohydrate metabolism. These repercussions not solely advance the accumulation of NSC but also refine nutritional composition and ultimately upgrade rice quality. This investigation offers invaluable perceptions into the modulation of rice quality through optimized water and fertilizer management, contributing to the sustainable advancement of rice production and accommodating the escalating demand for high-quality rice in the marketplace.

### 3.3. Effect of Water and Fertilizer on Lodging Resistance

The integration of water and fertilizer management is crucial in shaping the growth conditions of hybrid rice, thereby significantly influencing its lodging resistance. By ensuring an optimal supply of water and nutrients, this management approach effectively stimulates photosynthesis and carbohydrate biosynthetic processes [29]. The augmented photosynthesis leads to the accumulation of additional NSC, specifically soluble sugars and starches, which form the material basis for stem strength and durability [30,31].

The elevated NSC content not only enhances the fullness and thickness of the rice stem, resulting in a stronger morphological appearance, but also significantly boosts its physical resilience [32]. Furthermore, these NSC can potentially be converted to structural carbohydrates, such as cellulose and lignin. This conversion notably increases the mechanical strength of the stem, effectively improving its lodging resistance, a key factor in preventing lodging caused by natural elements like wind and rain [31,33].

Additionally, the combined approach of water and fertilizer management favorably impacts the morphological structure of rice stems [17]. Through meticulous regulation, it promotes thicker stem nodes and judicious adjustments in internode length [34]. These morphological enhancements significantly contribute to lodging resistance, allowing rice plants to maintain upright growth even in challenging environmental conditions, thus ensuring normal physiological functions and yield production [35,36].

In conclusion, the coordinated regulation of water and fertilizer management optimizes the growth environment for hybrid rice, boosting photosynthetic efficiency and carbohydrate accumulation. Moreover, it significantly improves the morphological structure and mechanical strength of the stem, laying a firm foundation for enhanced lodging resistance and consistent high yields. This strategy is vital in satisfying the demand for high-yield, high-quality, and stress-resistant rice varieties in contemporary agriculture, further promoting the sustainable progress of rice production.

### 3.4. Synergistic Regulation of Yield, Quality, and Lodging Resistance by Water and Fertilizer Management

**Figure 10:**
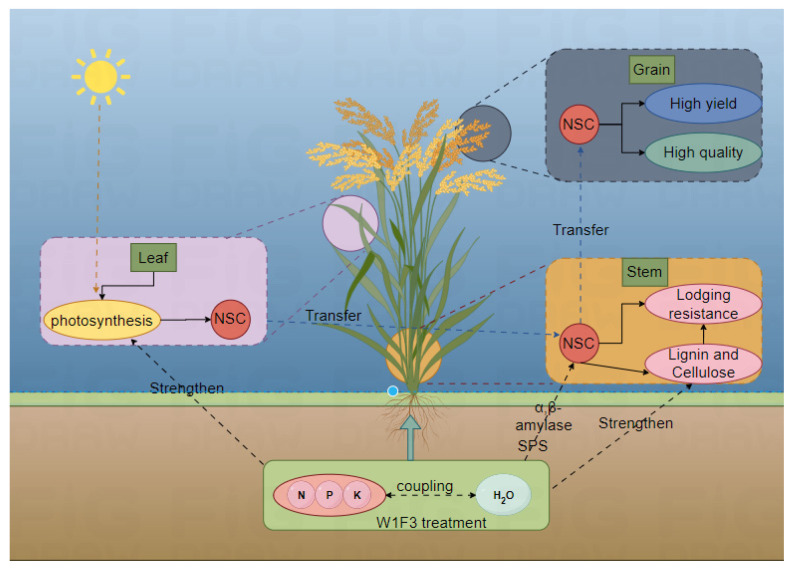
Synergistic regulation of yield, quality, and lodging resistance by water and fertilizer management.

The implementation of a carefully crafted water and fertilizer management strategy, notably the W1F3 treatment, has demonstrated remarkable efficacy in optimizing the growth environment for hybrid rice. As clearly depicted in Figure 10 created by Figdraw, this sophisticated strategy comprehensively regulates rice yield, quality, and lodging resistance via multiple mechanisms, wherein NSC assumes a pivotal function.

Initially, the optimized water and fertilizer management notably boosts the photosynthetic efficiency of leaves, facilitating significant NSC synthesis and accumulation. These NSC, including soluble sugars and starches, furnish essential energy for rice growth and development [37]. Simultaneously, they directly contribute to starch accumulation and protein synthesis within rice grains, laying a robust material foundation for high-yielding rice [38,39].

Furthermore, regarding yield formation, the ample NSC reserve expedites the rate of dry matter accumulation [24]. Augmented photosynthesis enables rice plants to more efficiently convert light energy into chemical energy, storing it in the form of NSC [40,41]. The precise regulation of carbohydrate production and transport further advances the grain filling process, thereby contributing to elevated yield [42,43].

Moreover, in terms of rice quality, the timely transfer and judicious distribution of NSC ensure uniform grain filling. This not only leads to fuller rice grains but also enhances the head rice rate and length–width ratio while diminishing chalkiness and chalky grain rate, significantly elevating the overall quality of rice [24,27].

Additionally, NSC accumulation plays a pivotal role in lodging resistance. The buildup of NSC augments the stem’s fullness and thickness, bolstering its visual sturdiness [30]. Furthermore, optimized water and fertilizer management fosters the conversion of certain NSC into structural carbohydrates, such as cellulose and lignin, through amplified activity of enzymes like SPS, α-amylase, and β-amylase [24,31]. These components considerably strengthen the mechanical robustness of the stem, thereby effectively enhancing its lodging resistance [44,45,46].

In conclusion, through the implementation of an optimized water and fertilizer management strategy, particularly the W1F3 treatment, comprehensive modulation of rice growth has been attained, with NSC functioning as the core regulator. This approach not only elevates photosynthetic efficiency but also promotes NSC accumulation and transport processes. More significantly, it exhibits remarkable impacts in synergistically optimizing rice yield, quality, and lodging resistance. This investigation not only reveals the intricate relationship between water and fertilizer management and NSC dynamics but also offers invaluable theoretical and practical insights for attaining high-yield and high-quality cultivation of hybrid rice, significantly contributing to the sustainable advancement of rice production.

## 4. Materials and Methods

### 4.1. Climatic Condition

In 2022, the average daily minimum and maximum temperatures were notably higher than those recorded in 2023. Conversely, the average daily rainfall was significantly lower in 2022 compared to 2023. While temperatures exhibited an upward trajectory from the transplanting to flowering stages in both years, rainfall patterns were distinct. Specifically, rainfall was markedly more concentrated during the Transplanting to Panicle Initiation phase in 2022, and from Panicle Initiation to Flowering in 2023, as detailed in Table 3.

### 4.2. Experiment Design and Plant Materials

The experiments were conducted in rice paddies located in Oujiangcha Village (geographic coordinates: 28°29′55″ N, 112°35′59″ E; altitude: 12 m above sea level), Yiyang City, Hunan Province, during 2022 and 2023. The experimental design employed a split-split plot design, focusing on three primary factors: rice variety (V), water management (W), and nitrogen fertilizer application (F). The study encompassed 72 split plots, each with an area of 20 m^2^, totaling an experimental area of 1440 m^2^ (excluding water channels). The soil had the following properties: pH 5.1, 34.8 g kg^−1^ organic matter, 1.6 g kg^−1^ total nitrogen (N), 13.8 mg kg^−1^ Olsen phosphorus (P), and 103.4 mg kg^−1^ exchangeable potassium (K). The soil test was based on samples taken from the upper 20 cm of the soil before the application of basal fertilizers in 2022.

**Figure 11:**
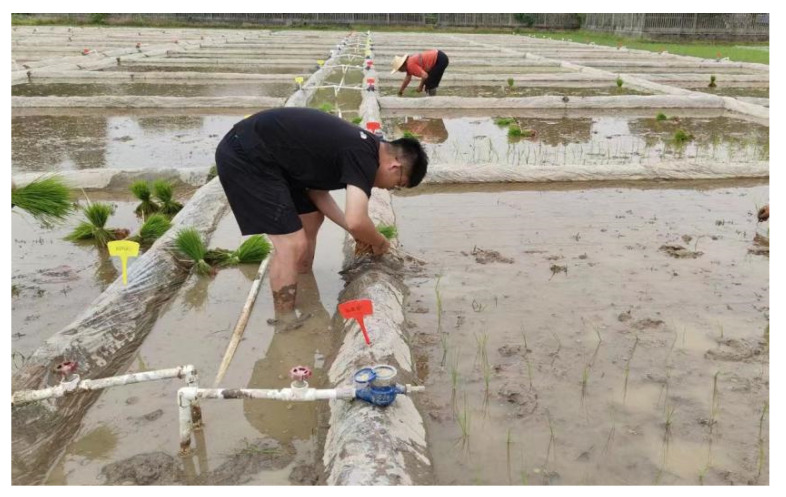
Water pipes and water meters in the community.

The main plots were assigned different water treatments (W), categorized into three levels: W1—continuous wet-shallow irrigation throughout the entire growth cycle, W2—conventional flooded irrigation, and W3—thin, shallow, wet, and dry irrigation. The specific water layer control standards for these irrigation methods are summarized in Table 4. All irrigation treatments were manually administered, with precise measurements of the irrigation volume using water meters (refer to Figure 11). The split plots were designated for evaluating different rice varieties (V), specifically as follows.

This study employed a split-split plot experimental design, involving three factors: cultivar, water, and nitrogen fertilizer. The main plots focused on water treatment (W), with three levels: W1 represents continuous wet-shallow irrigation throughout the entire growth cycle, W2 stands for flooded irrigation (the conventional irrigation method used by large-scale grain farmers), and W3 signifies thin, shallow, wet, and dry irrigation (the typical irrigation approach adopted by small-scale grain farmers). Detailed water layer control standards for these three irrigation modes are outlined in Table 4. The split plots concentrated on cultivar (V), comprising two levels: Xinxiangliangyou 1751 (V1, XXLY1751), sourced from Hunan Jinse Nonghua Seed Industry Technology Co., Ltd. (Changsha, China), and Yueliangyou Meixiangxinzhan (V2, YLYMXXZ), provided by Yuan Longping High-Tech Agriculture Co., Ltd. (Changsha, China)—both widely grown rice in the middle and lower Yangtze River. The split-split plots investigated nitrogen application rates (N), divided into four levels: F1, F2, F3, and F4, corresponding to pure nitrogen applications of 0, 180, 225, and 270 kg ha^−1^, respectively. The fertilizer adopted the multiple fertilization method, with the nitrogen fertilizer (urea) application ratio of base fertilizer: tiller fertilizer: ear fertilizer being 5:3:2. The base fertilizer, tiller fertilizer, and ear fertilizer were applied 1–2 days before transplanting, 10 days after transplanting, and at the beginning of young panicle differentiation (jointing stage), respectively. The fertilization ratio uniformly adopted an NPK ratio of N:P_2_O_5_:K_2_O = 1:0.6:1.2. Phosphate fertilizer (P_2_O_5_, 16%) was entirely used as base fertilizer, while potassium fertilizer (K_2_O, 60%) was split evenly between the base fertilizer and the tiller fertilizer. Before transplanting, equal amounts of phosphate and potassium fertilizers were applied as base fertilizers to supplement the nutritional elements required by rice across all treatments. The experiment comprised 24 treatments, with separate isolation between plots to ensure independent drainage and irrigation. There were three replicates, arranged randomly according to a split-plot design, resulting in a total of 72 split plots. Each split plot covered an area of 20 m^2^, and the total experimental area was 1440 m^2^ (excluding water channels). Disease, pest, and weed control were strictly managed according to high-yield cultivation practices.

### 4.3. Sampling and Measurement

**Photosynthetic rate** Photosynthetic rates were measured using a portable photosynthesis measurement system LI-6800 (LI-COR, Inc., Lincoln, NE, USA) at the tillering stage (selecting the second fully expanded leaf from the top), booting stage, full heading stage, and milk-ripe stage (selecting the flag leaf). Measurements were conducted on clear, cloudless, and windless days between 9:00 and 12:00. Two representative plants were selected from each plot, and each plant was measured three times. The average value of these measurements was taken as the final result [47].

**Total dry weight (TDW)** Sampling was conducted during the tillering stage, booting stage, full heading stage, and filling stage. Ten clusters were consecutively selected from each plot, and the stems, sheaths, leaves, and panicles were separated and packaged. Samples were oven-dried at 105 °C for 30 min to stop enzyme activity and then dried to a constant weight at 80 °C [48].

**Yield** Yield was determined by harvesting and drying grains from each plot separately. The actual yield was calculated based on a moisture content of 14% in the rice grains [49].

**Appearance Quality** Appearance quality measurements, including chalkiness degree and grain length–width ratio, were conducted using a Wanshen SC-E rice appearance quality detector (Wanshen Testing Technology Co., Ltd., Hangzhou, China) and its analytical system [50].

**Nutritional Quality Amylose** content was determined using the iodine-blue colorimetric method following the Ministry of Agriculture standard NY/T 2639-2014. Protein content was measured by digesting polished rice flour with H_2_SO_4_-H_2_O_2_ and using Nessler’s colorimetric method to determine nitrogen content. Protein content was calculated by multiplying nitrogen content by a conversion factor of 5.95 [51].

**Lodging index** Twenty uniformly growing main stalks were randomly selected from each plot 25 days after heading. The third, fourth, and fifth internodes from the base were cut off, and their lengths were measured. Bending resistance at the midpoint of these internodes was determined using a YYD-1 stalk strength tester (Tuopu Instruments Co., Ltd., Hangzhou, China), with a support span of 5 cm between the two fulcrums. Various mechanical parameters were calculated using established formulas [52].

The breaking bending moment (M, g·cm) = F × L/4

where F is the force applied when the measured basal section is broken, with the unit of kg; L is the distance between the two supports, with the unit of cm.

The bending moment (WP, g·cm) = SL × FW

where SL is the distance from the base of the internode to the top of the panicle, with the unit of cm; FW is the fresh weight from the base of the internode to the top of the panicle, with the unit of g.

The lodging index (LI) = WP/M × 100%

**NSC** Concentrations of NSC (soluble sugars and starch) in straw were determined at the heading stage, grain filling stage, and maturity stage, following the method outlined by [53]. Aliquots from the soluble sugars and starch extracts were assayed using anthrone reagent colorimetry, employing a spectrophotometer (L8, Shanghai Yidian Analytical Instrument Co., Ltd., Shanghai, China) set at a wavelength of 620 nm. Starch content was calculated by multiplying the glucose level by a conversion factor of 0.9. NSC concentration was estimated by summing the concentrations of soluble sugars and starch.

**Amylase** The detection of α-amylase, β-amylase, and sucrose phosphate synthase (SPS) was conducted using ELISA kits. The detection results were measured in terms of absorbance (OD value) at a wavelength of 450 nm using the DNM-9606 microplate reader (Beijing Perlong New Technology Co., Ltd., Beijing, China). With the OD value plotted on the vertical axis (Y) and the corresponding standard concentration of the analyte on the horizontal axis (X), a standard curve was constructed. By referencing the OD value of the sample against this standard curve, the concentration of the analyte in the sample could be determined.

**Lignin and Cellulose** The lignin and cellulose content measurements were described by [54].

### 4.4. Statistical Analysis

The field experiment employed a 3 × 4 × 2 factorial design, comprising three water levels, four fertilizer levels, and two varieties for each treatment combination within three blocks. We utilized a mixed-effects analysis of variance for all variables in our study. The fixed effects included water, fertilizer, variety, year, and the interaction between water and fertilizer. We regarded blocks as a random effect. The data were analyzed using analysis of variance (ANOVA, SAS Version 9.1.2, Cary, NC, USA). The means of the water and fertilizer treatments for each variety were compared using the least significant difference test (LSD) at a 0.05 probability level. Graphs were constructed using Microsoft Excel 2017 (Microsoft Corp., Redmond, USA).

## 5. Conclusions

This study found that the W1F3 treatment (wet-shallow irrigation and appropriate fertilization (N-P_2_O_5_-K_2_O: 225–135–270 kg ha^−1^)) had a significant positive impact on hybrid rice. Specifically, the effects are manifested in the following ways: (1) The yield of hybrid rice was significantly enhanced by improving photosynthetic efficiency, promoting dry matter accumulation, and optimizing the dynamics of non-structural carbohydrates (NSC). (2) The effective increase in NSC accumulation and improvement in carbohydrate metabolism resulted in an increased rate of head rice, an increased grain length/width ratio, and a reduction in chalkiness and the chalky grain rate. (3) By promoting photosynthesis, increasing NSC accumulation, enhancing stem fullness and thickness, and facilitating the conversion of NSC to structural carbohydrates such as cellulose and lignin, the mechanical strength of the stems was significantly improved, thereby enhancing the lodging resistance of hybrid rice. In summary, the W1F3 treatment comprehensively regulated the yield, quality, and lodging resistance of hybrid rice through the key role of NSC. This reveals a close connection between water and fertilizer management and NSC dynamics, providing an important basis for achieving high-yield and high-quality cultivation of hybrid rice. It has positive implications for promoting the sustainable development of rice production.

## Figures and Tables

**Table 1:** Irrigation and fertilization treatments effect on the lodging resistance of different rice stem internodes in 2022 and 2023. J3 and J4 represent the third and fourth basal stem internodes, respectively.

Year	Variety	Irrigation	Fertilizer	M		WP		LI	
				J3	J4	J3	J4	J3	J4
2022	V1	W1	F1	861.99 e	833.07 g	1159.26 bcd	923.69 def	0.74 de	0.9 f
			F2	955.67 c	932.54 de	1475.44 a	1174.63 a	0.65 g	0.79 g
			F3	1008.96 b	1055.43 a	1545.99 a	1176.86 a	0.65 g	0.9 f
			F4	999.54 b	761.54 h	1069.37 de	1125.94 ab	0.93 b	0.68 h
		W2	F1	717.56 g	757.67 h	1024.27 de	769.23 gh	0.7 f	0.98 e
			F2	742.9 fg	947.31 cd	1218.76 bc	915.86 def	0.61 h	1.03 d
			F3	982.38 bc	1005.86 b	1120.87 cde	834.78 fg	0.88 c	1.2 b
			F4	1005.08 b	983.44 bc	1013.36 e	699.08 h	0.99 a	1.41 a
		W3	F1	769.54 f	880.81 f	1064.27 de	990.99 cd	0.72 ef	0.89 f
			F2	981.74 bc	895.71 ef	1290.56 b	1038.12 bc	0.76 d	0.86 f
			F3	904.99 d	884.15 f	1222.8 bc	932.08 de	0.74 de	0.95 e
			F4	1053.27 a	927.14 def	1029.34 de	852.12 efg	1.02 a	1.09 c
	Mean			915.30	905.39	1186.19	952.78	0.78	0.97
	V2	W1	F1	460.05 e	623.43 d	1134.24 f	882.62 de	0.41 d	0.71 cd
			F2	465.03 e	598.32 d	1520.39 a	1130.49 a	0.31 g	0.53 g
			F3	551.55 b	708.63 b	1422.99 ab	1073.58 ab	0.39 d	0.66 f
			F4	599.2 a	758.58 a	1212.07 def	1030.63 abc	0.49 a	0.74 c
		W2	F1	454.6 e	627.8 d	1008.7 g	764.52 f	0.45 c	0.82 b
			F2	460.53 e	697.12 bc	1373.79 bc	1027.06 abc	0.34 f	0.68 ef
			F3	463.35 e	679.84 bc	1249.3 de	928.85 cd	0.37 e	0.73 c
			F4	515.49 d	745.11 a	1168.81 ef	820.59 ef	0.44 c	0.91 a
		W3	F1	451.34 e	625.83 d	1027.19 g	784.05 ef	0.44 c	0.8 b
			F2	529.81 cd	674.48 c	1417.64 b	1043.46 ab	0.37 e	0.65 f
			F3	541.7 bc	749.42 a	1280.45 cd	1016.42 bc	0.42 cd	0.74 c
			F4	558.12 b	620.01 d	1174.3 ef	886.62 de	0.48 b	0.7 de
	Mean			504.23	675.71	1249.16	949.07	0.41	0.72
2023	V1	W1	F1	895.28 g	1078.83 f	1401.65 bc	1070.58 de	0.64 g	1.01 d
			F2	1082.66 e	1271.38 bc	1577.03 a	1206.32 b	0.69 fg	1.05 d
			F3	1239.04 bcd	1327.3 abc	1573.71 a	1299.14 a	0.79 de	1.02 d
			F4	1317.46 ab	1301.19 abc	1382.95 bc	1161.1 bc	0.95 c	1.12 d
		W2	F1	867.32 g	1134.01 def	1095.14 d	867.17 g	0.79 de	1.31 c
			F2	884.6 g	1359 ab	1302.8 c	990.76 e	0.68 fg	1.37 bc
			F3	1202.37 cd	1415.9 a	1267.95 c	954.06 ef	0.95 c	1.48 ab
			F4	1263.2 abc	1223.3 cde	1110.95 d	895.99 f	1.14 a	1.37 bc
		W3	F1	994.43 f	1115.41 ef	1340.03 bc	984.98 e	0.74 ef	1.13 d
			F2	1040.69 ef	1259.89 bcd	1473.39 ab	1108.15 cd	0.71 f	1.14 d
			F3	1173.33 d	1373.67 ab	1403.52 bc	1006.83 e	0.84 d	1.36 bc
			F4	1338.62 a	1415.28 a	1279.68 c	901.6 f	1.05 b	1.57 a
	Mean			1108.25	1272.93	1350.73	1037.22	0.83	1.24
	V2	W1	F1	599.5 e	773.81 d	1240.13 de	822.6 g	0.48 bc	0.94 ab
			F2	682.76 cd	841.56 bc	1616.17 a	1153.71 a	0.42 e	0.73 ef
			F3	701.6 bc	877.17 bc	1600.53 a	1107.21 ab	0.44 de	0.79 d
			F4	757.63 a	944.24 a	1473.88 b	1050.86 bcd	0.51 b	0.9 bc
		W2	F1	524.99 f	705.23 e	1128.8 e	940.1 ef	0.47 cd	0.75 def
			F2	640.9 de	851.91 bc	1380.83 bc	1101.69 abc	0.46 cd	0.77 de
			F3	663.18 cd	992.58 a	1319.2 cd	1015.98 cde	0.5 b	0.98 a
			F4	740.67 ab	831 bcd	1298.16 cd	921.32 f	0.57 a	0.9 bc
		W3	F1	543.63 f	699.8 e	1298.25 cd	1007.25 def	0.42 e	0.69 f
			F2	696.26 bc	881.03 b	1606.12 a	1122.51 ab	0.43 de	0.78 de
			F3	732.91 ab	1000.65 a	1458.66 b	1016.52 cde	0.5 b	0.98 a
			F4	770.8 a	815.84 cd	1304.82 cd	936.19 ef	0.59 a	0.87 c
	Mean			671.24	851.24	1393.80	1016.33	0.48	0.84

Within a column for each year, means followed by the same letters are not significantly different according to LSD (0.05). M, breaking bending moment; WP, bending moment; LI, lodging index.

**Table 2:** Fertilizer, irrigation, variety, and year affect Pn (net photosynthetic rate), TDW (total dry weight of the ground part), yield, HRY (head rice rate), protein, amylose, NSC, cellulose, lignin, and BR (bending resistance force).

SOV	Pn	TDW	Yield	HRY	Protein	Amylose	NSC	Cellulose	Lignin	BR
F	**	**	**	**	**	**	**	**	**	**
V	**	**	**	**	**	**	**	**	**	**
W	**	**	*	**	**	**	**	**	**	**
Y	**	**	**	*	**	**	**	**	NS	**
F*W	*	**	**	NS	NS	**	**	**	NS	*
F*W*V	NS	**	NS	NS	NS	NS	NS	NS	NS	NS
F*W*Y	NS	NS	NS	NS	NS	**	NS	NS	NS	NS
F*V*W*Y	NS	**	NS	NS	NS	NS	NS	NS	NS	NS

Significant treatment effects within a main category are denoted by NS (*p* > 0.05) or * (0.01 < *p* ≤ 0.05) or ** (*p* ≤ 0.01). Pn, net photosynthetic rate; TDW, total dry weight of the ground part; HRY, head rice rate; BR, bending resistance force.

**Table 3:** The climate conditions by rice growth stages in 2022 and 2023.

Year	Min T	Max T	ADR
	Transplanting to Panicle initiation		
2022	26.3	34.2	11.2
2023	25.9	32.1	5.1
		Panicle initiation to Flowering	
2022	28.2	37.7	0.1
2023	26.1	34.5	6.9
		Flowering to Maturity	
2022	22.6	31.6	0.1
2023	21.2	29.8	0.4
		Transplanting to Maturity	
2022	25.8	34.6	3.3
2023	24.4	32.2	4.2

Average daily minimum temperature (Min T, °C), average daily maximum temperature (Max T, °C), and average daily rainfall (ADR, mm day^−1^) for each growing period.

**Table 4:** Thresholds of water level for different rice irrigation modes (mm).

Irrigation	RS	ES	LS	JS	HS	GS	MS
W1	0–20–30	0–20–30	SF	0–20–30	0–20–30	0–20–30	NF
W2	20–50–70	20–50–70	SF	20–50–70	20–50–70	20–50–70	NF
W3	10–30–50	10–30–50	SF	10–40–60	10–40–60	10–20–40	NF

Three continuous data in the table (e.g., 0–20–30, mm) represent the lower and upper limits of irrigation and the maximum limit after rain, respectively; RS represents the returning green stage, ES represents the early tillering stage, LS represents the late tillering stage, JS represents the jointing and booting stage, and HS represents the heading stage. GS represents the grain-filling stage, and MS represents the maturity stage. SF represents sun-dried field, NF represents natural drying.

## Data Availability

The data presented in this study are available upon request from the corresponding author.
